# Engaging tribal communities for improving people’s health: lessons from rural Melghat, India

**DOI:** 10.7189/jogh.15.03020

**Published:** 2025-06-27

**Authors:** Satav Ashish, Satav Kavita, Dani Vibhawari, Raje Dhananjay, Khirwadkar Shubhada, Fernandes Genevie, Pande Vitthal, Palaskar Manik, Jambekar Sukarai

**Affiliations:** 1 MAHAN Trust, Dharni, Amaravati, Maharashtra, India; 2 Usher Institute, University of Edinburgh, Respire, Edinburgh, UK

## Abstract

Tribal communities constitute around 9% of India’s population, facing severe socioeconomic and health disparities. High maternal and infant mortality, adult mortality, malnutrition, infectious and non-communicable diseases, and addiction pose significant challenges. Limited health care access, long distances, inadequate infrastructure, and cultural reliance on traditional medicine exacerbate poor health outcomes. MAHAN Trust has addressed these challenges in the remote Melghat region since 1997 through culturally sensitive, community-driven health care interventions. Ethical and participatory community engagement has significantly reduced malnutrition and mortality in Melghat. These scalable, low-cost strategies can be adapted to improve tribal health outcomes in other low-resource settings globally.

Tribal people constitute around 9% of the population in India, accounting for 104 million people across 705 tribes (Figure S1 in the **Online Supplementary Document**) [2]. Most tribal populations live in Madhya Pradesh, Maharashtra, Odisha, Jharkhand, Chhattisgarh, and Rajasthan. Nearly nine in 10 tribals live in rural parts of the country, predominantly in hilly and forested areas. While the tribal population in India is heterogeneous, they commonly face socioeconomic and health challenges. Close to 40% of tribals live below the poverty line, which equals spending by one person less than INR 27 (*i.e.* GBP 0.25) per day [2].

Tribal communities lag the national average on several health indicators. They account for over 50% of all maternal and infant deaths in the country [2]. Two-thirds of tribal women suffer from anaemia, 80% of females in Melghat are anaemic, only 15% of pregnant women complete antenatal care visits, and nearly 30% still deliver at home in tribal areas of India. Only 56% of tribal children receive full immunisation, 77% are anaemic, and >55% are underweight [3,4]. The tribal population faces a quadruple burden of disease: malnutrition, infectious and non-communicable diseases, and mental health illness, particularly addiction. More than 72% of tribal men use tobacco, and >50% consume alcohol [5].

Access to and use of public health care services and health care-seeking behaviours among tribal people are poor [2]. Long distance, weak road infrastructure, and limited transportation make reaching primary health care centres and hospitals difficult. There is a significant shortfall in public health care specialists and the general workforce in rural and remote areas of the country, where most of the tribal population resides. Further, tribal communities also rely heavily on traditional beliefs and practices compared to allopathic treatment, and public health care services may not always be attuned to these contextual norms, values, and practices [6].

Health policies, programmes, and research to improve the health outcomes of the tribal populations in the country need to be tailored and contextualised. More importantly, tribal members need to be involved, meaningfully engaged and represented in the prioritisation, planning and delivering any intervention for them [7]. The MAHAN Trust is a non-governmental organisation that has provided hospital- and community-based health services and programmes for tribal populations in rural Melghat since 1997 [8]. Melghat is a difficult-to-reach, hilly, forested area in Maharashtra, with 3 00 000 people across 320 villages, of which >80% are tribal. The diverse range of tribal groups, each with their distinct beliefs, norms and practices, with high rates of chronic health challenges, poor infrastructure and weak links with the mainstream health system, makes the situation in Melghat uniquely challenging compared to other tribal areas in the country. In this viewpoint, we discuss the key lessons learned in community engagement over two decades of MAHAN Trust’s experience working with tribal communities across several health programmes and research projects covering malnutrition, child health, adult health, respiratory health and de-addiction (Figure S2 in the **Online Supplementary Document**).

## BEST PRACTICES

### Listen to the community and understand their priorities

**Figure 1:**
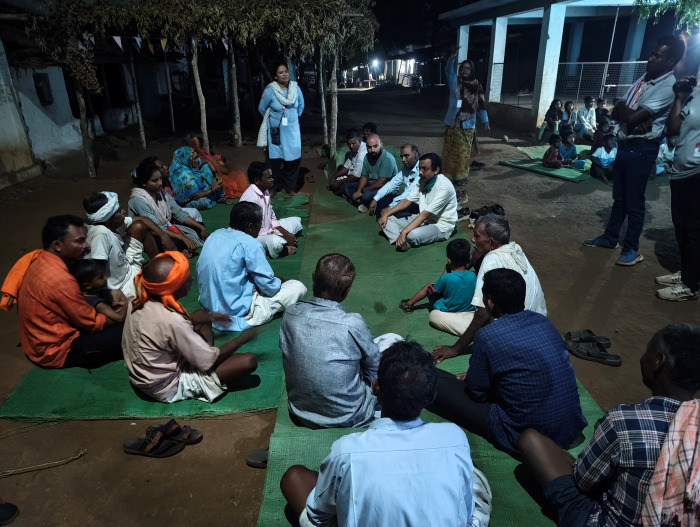
*Gramsabha*-community meeting for the improvement of community engagement in the MAHAN programme. Permission was obtained from the members of *Gramsabha* to start any programme and solve their problems under PESA act. Authors are part of the team.

A village is the smallest administrative unit in India, and every village has a *Gram Panchayat* or a local governance unit, which convenes at *Gramsabhas* or meetings attended by 60% of the adult population [9]. The MAHAN team arranged *Gramsabhas* regularly (**Figure 1**) and listened to the communities to understand their problems. Malnutrition and mortality emerged as their biggest concerns, and they wanted free, easily accessible, and culturally acceptable services delivered by those who understood their local dialect and customs. Community members were then involved in designing culturally acceptable interventions, such as home-based child/adult care, which was delivered by village health workers selected by *Gramsabhas* [10].

### Respect local norms and build trust

The two founders of MAHAN Trust relocated from a city to rural Melghat. They lived in a hut from which they ran the first hospital, gradually influencing community acceptance and participation. Traditional faith healers, influential community stakeholders, were allowed into the hospital's intensive care unit to practice rituals around the patients. Meetings are typically conducted after work hours in the evenings, as most community members work on farms during the day. The MAHAN team always sits on the floor with community members in all meetings. Embedding such practices in routine interactions with the community can assure and demonstrate respect and sensitivity for tribal traditions and norms.

Early on, the team mapped and identified influential gatekeepers in the villages, including the *sarpanch* (*i.e.* village leader), traditional faith healers, police *patil*, *adya patel* (*i.e.* local law enforcement selected by traditional community members), schoolteachers and traditional birth attendants. Regular and transparent communication, participation in local community events, empathetic listening, and support helped to build relationships over time, and these gatekeepers have facilitated community trust in the MAHAN team and endorsed their participation in health and social development programmes. Community members consenting to autopsy (typically culturally unacceptable) for a study on minimally invasive tissue sampling of infants and adults to ascertain the cause of death was a powerful example of trust [11].

### Seek consent and approval from the community

Realising the need for home-based healthcare services to address rising rates of malnutrition, child mortality, and adult mortality, the team worked with community members to select village-level female health workers called *arogyadoot* (*i.e.* messengers of good health) who lived in the same area, spoke the tribal dialect of *Korku*, and were familiar with the local traditions, norms, and practices. Similarly, the community identified youth volunteers or *yuvadoots* (*i.e.* youth messengers) to work on interventions addressing social issues such as addiction.

The MAHAN team keeps community members appraised of all the ongoing interventions through *Gramsabha* meetings. It discusses any planned programmes for their consent and input. No programmatic or research intervention commences without the permission of the tribal leaders and the whole village, where it will be delivered. As literacy levels are low, the team organises video consent for research study participants, recorded by a tribal leader or a trusted village stakeholder.

### Involve stakeholders throughout, using culturally acceptable ways

The team identifies and prioritises community stakeholders relevant to specific health programmes and research studies and involves them throughout all stages. For recent studies on developing a community-based tuberculosis intervention and conducting photovoice for chronic respiratory disease as part of the NIHR Global Health Research Unit on Respiratory Health, a range of community and primary health care stakeholders were invited to a stakeholder advisory panel, which will be consulted throughout the research stages.

Tribal communities have a rich song, dance, arts, and crafts heritage. Health messages were communicated through these cultural channels, leading to greater acceptance and adherence by community members. Songs in the local dialect, *bhajans* (*i.e.* devotional songs), community yoga, word of mouth, and *davandi* (*i.e.* drum beating) were used to convey health messages. Young children have been promoting healthy habits through *prabhat pheri* (*i.e.* village walks). Folk plays (*i.e. Kham*) and a documentary film on malnutrition, deaths, and alcohol addiction featuring local youth were presented across villages using mobile vans. An inter-village volleyball competition mobilised and engaged youth, all of which contributed to the *Gramsabha* passing a resolution banning the production and consumption of alcohol in villages, youth giving up social drinking, and participation in residential de-addiction camps.

### Adopt a culture of using evidence and continuous learning

The team relies on periodic information gathering through surveys, interviews, group discussions, community meetings, and rigorous research studies around the trends in population health, barriers and facilitators for health behaviours, and the implementation and effectiveness of the various health interventions [10]. The generated evidence is then shared with community members and informs the development of new initiatives and training programmes for frontline health workers, traditional birth attendants, and counsellors.

### Develop culturally acceptable solutions through ethical community engagement

Community engagement in research is often associated with achieving goals such as recruitment or retention, but ethical engagement also calls for reciprocity and requires benefits for the community stakeholders. Besides providing health care services, the MAHAN team informs villagers about ongoing government schemes and subsidies and helps them avail themselves of these benefits. The team also advocated for the successful transfer of police personnel involved in an incident of violence against tribal members.

The team developed community-based management of severe malnutrition to address high malnutrition rates that local village health workers delivered. Culturally accepted snacks and meals, fortified with nutrients and developed in consultation with community members, are now widely accepted and consumed in the villages. Local women are employed to prepare these snacks/meals in a hygienic and standardised way. Live household demonstrations are organised to support families in preparing local nutritious foods. Kitchen gardens are also promoted as another sustainable solution for producing and consuming nutrient-rich foods.

## CONCLUSIONS

Engaging tribal communities in health and development initiatives can be challenging due to language, literacy, socioeconomic, cultural, and geographic barriers. Being ethical, safe, and cost-effective, these community engagement practices can be adapted and scaled up in similar difficult-to-access tribal areas of India and other low- and middle-income countries through training, and importantly, institutionalising community engagement within the practices of government, non-government and other stakeholders working in public health. MAHAN Trust’s experience has shown that, although arduous and time-consuming, respect, trust, reciprocity, and relationships can go a long way in successfully engaging, partnering with and ultimately empowering tribal communities for the improvement of their health care-seeking behaviours and outcomes; these practices have contributed to a significant reduction in severe malnutrition [12,13] and mortality [10].

## Additional material


Online Supplementary Document
