# Supplementary material for: Engaging tribal communities for improving people’s health: lessons from rural Melghat, India
Source: J Glob Health. 2025 Jun 27;15:03020. doi: 10.7189/jogh.15.03020 (PMC12201932; doi:10.7189/jogh.15.03020)
Supplement: Online Supplementary Document [file jogh-15-03020-s001.pdf]

Supplement to: Satav A, Satav K, Dani V, Raje D, Khirwadkar S, Fernandes G, Pande V, Palaskar M, Jambekar S. Engaging tribal communities for improving people's health: lessons from rural Melghat, India. J Glob Health. 2025;15:03020.

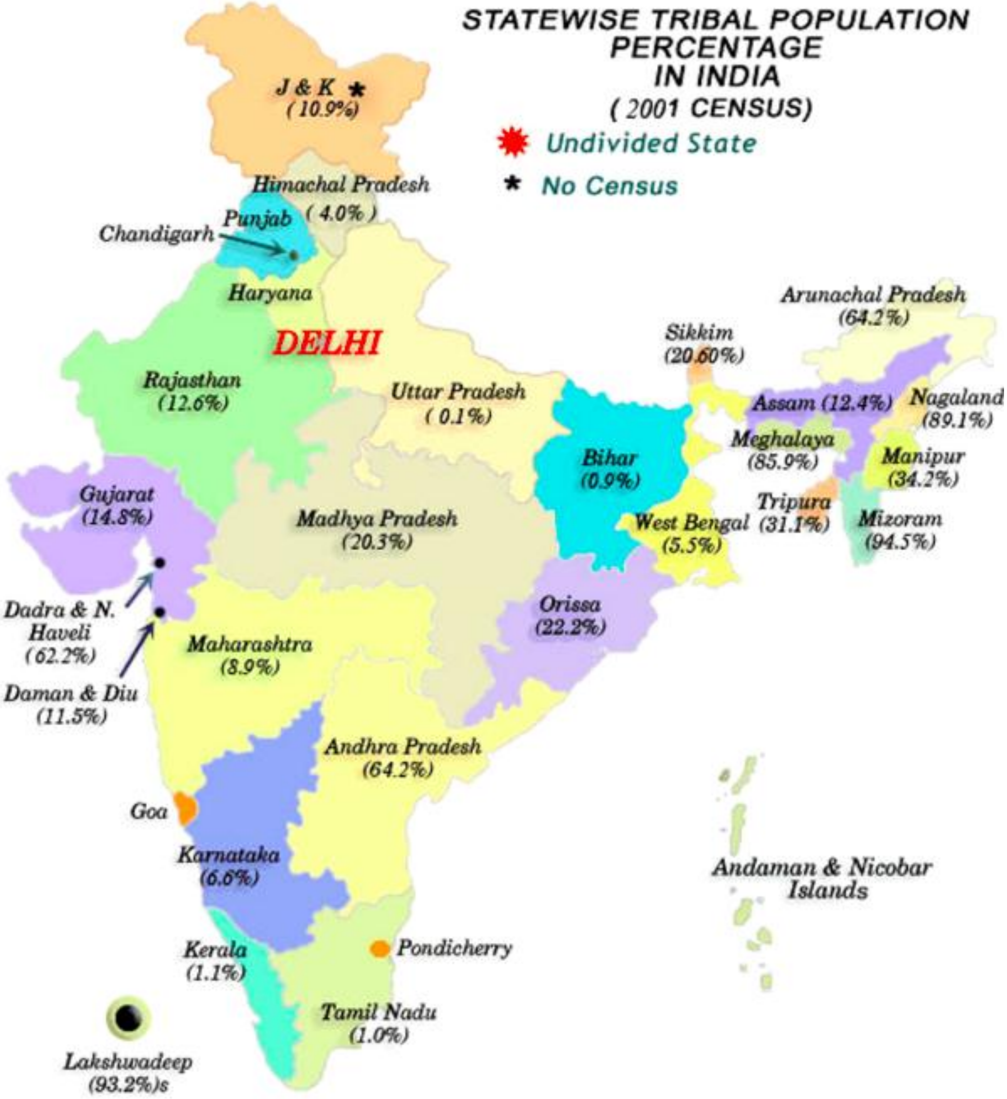

Figure S1. Map of state-wise tribal population percentage in India, 2001, adapted from [1].

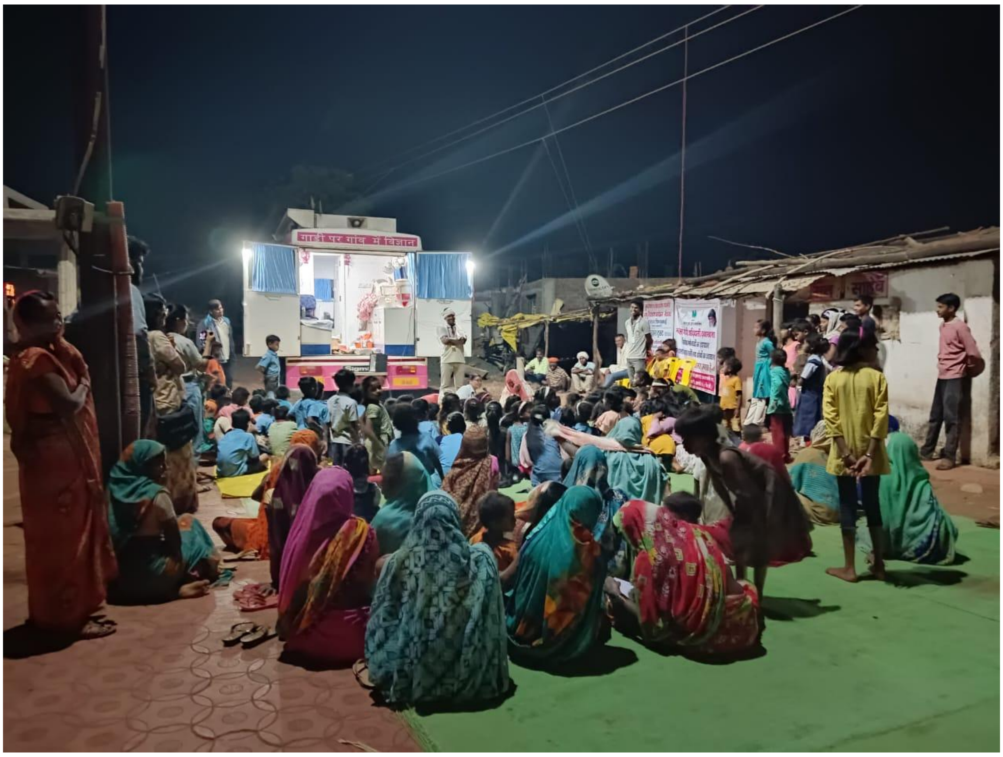

Figure S2. Behaviour change communication through an audio-visual show to address tribal health issues. Permission to use obtained from the MAHAN Trust, of which the authors are members, under the PESA Act of Tribal Rights related to the local *gramsabha*.

REFERENCES

1 Mohindra KS, Labonté R. A systematic review of population health interventions and Scheduled Tribes in India. BMC Public Health. 2010;10:438. Medline:20659344 doi:10.1186/1471-2458-10-438
